# Microbial Oncotarget: Bacterial-Produced Butyrate, Chemoprevention and Warburg Effect

**DOI:** 10.18632/oncotarget.915

**Published:** 2013-02-28

**Authors:** Dallas R. Donohoe, Kaitlin P. Curry, Scott J. Bultman

**Affiliations:** Department of Genetics and Lineberger Comprehensive Cancer Center, University of North Carolina, Chapel Hill, NC, USA; Department of Genetics and Lineberger Comprehensive Cancer Center, University of North Carolina, Chapel Hill, NC, USA; Department of Genetics and Lineberger Comprehensive Cancer Center, University of North Carolina, Chapel Hill, NC, USA

The Human Microbiome Project is using next-generation sequencing and metagenomics to characterize microbial communities that inhabit our gastrointestinal tract and other body sites [1, 2]. Based on these efforts, it is becoming increasingly clear that commensal microbiota play a significant role in shaping human health and disease. Yet it will be necessary to identify important microbial metabolites and to understand how they regulate host biology. Butyrate is a short-chain fatty acid produced by bacterial fermentation of dietary fiber in the colon. Previous studies have demonstrated that colonocytes from germfree mice, which lack microbiota and butyrate, proliferate less and are in an energy-deprived state compared to colonocytes from conventionally-raised control mice [3, 4]. Cell proliferation and energy metabolism were restored when germfree mice were colonized with a butyrate-producing bacterium or when butyrate was provided directly through the diet. These results indicate that the microbial metabolite butyrate maintains colonocyte homeostasis. Intriguingly, although butyrate promotes proliferation of normal colonocytes, it has the opposite effect on cancerous cells where it inhibits cell proliferation and also induces apoptosis [5]. However, the mechanistic basis for butyrate having opposite effects on normal and cancerous cells is so poorly understood that it has been referred to the butyrate paradox.

A recent study by Donohoe et al. now provides considerable mechanistic insight by demonstrating that a fundamental difference in energy metabolism between normal and cancerous colonocytes can explain the butyrate paradox [6]. Normal colonocytes utilize butyrate as their preferred energy source, which as a fatty acid undergoes oxidative metabolism in the mitochondria. This was shown to underlie the ability of butyrate to stimulate normal colonocyte proliferation (Fig. 1A). In contrast, due to the Warburg effect, cancerous colonocytes become addicted to glucose and undergo high levels of glycolysis with relatively little mitochondrial oxidative metabolism. As a result, butyrate was not metabolized to the same extent in cancerous colonocytes, accumulated in the nucleus, and functioned as a histone deacetylase (HDAC) inhibitor to regulate genes that inhibited cell proliferation and promoted apoptosis (Fig. 1B). An important aspect of this study was the ability to prevent the Warburg effect from occurring in cancerous colonocytes by performing RNAi to deplete an important mediator of the Warburg effect (LDHA) or by growing the cancer cells in low-glucose conditions (which forced them to use glutamine as their primary energy source and undergo mitochondrial oxidative metabolism). Both experimental approaches resulted in butyrate stimulating cancer cell proliferation (Fig. 1C), which resembled normal cells rather than the same cancer cells when they underwent the Warburg effect. This ability of butyrate to promote cell proliferation was restricted to relatively low doses (0.5-1 mM). Higher doses of butyrate (2-5 mM) decreased cell proliferation and induced apoptosis in both normal colonocytes and cancerous colonocytes regardless of the Warburg effect. This can be explained by previous observations that 1-2 mM corresponds to the oxidative capacity of these cells [7]. Therefore, at concentrations greater than 2 mM, butyrate accumulates and functions as a HDAC inhibitor in normal colonocytes as well as cancerous colonocytes. It should be emphasized that butyrate is such an abundant metabolite in the lumen of the colon that a 0.5-5 mM dose range is physiologically relevant.

**Figure:**
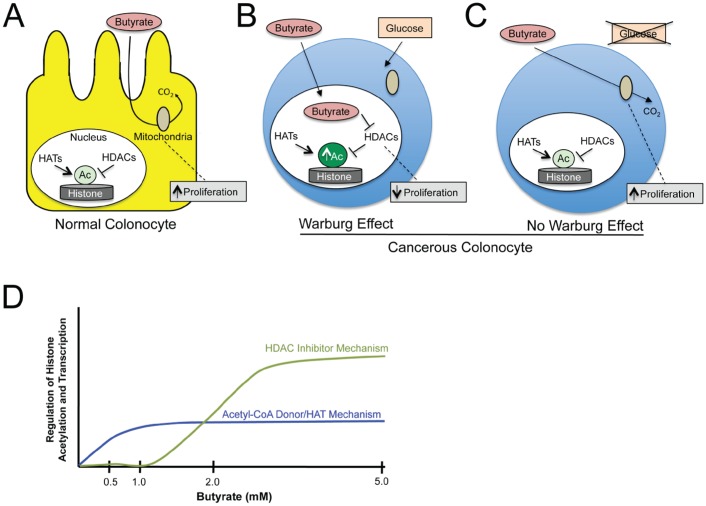


This study also demonstrated that the role of butyrate in epigenetics is more complicated than previously appreciated. In addition to functioning as an HDAC inhibitor, which was already known, butyrate can also increase histone acetylation by increasing histone acetyltransferase (HAT) activity. When butyrate is metabolized oxidatively, it contributes to acetyl-CoA production not only in the mitochondria but also in the cytosol and nucleus where it serves as an essential co-factor and acetyl-group donor for HATs. This pathway is dependent on the enzyme enzyme ATP citrate lyase (ACL) [8], and RNAi depletion of ACL was used to determine the relative importance of the acetyl-CoA/HAT and HDAC inhibition mechanisms. Consistent with the oxidative metabolic capacity of the cells being studied, the predominant mechanism was acetyl-CoA/HAT at 0.5-1 mM but shifted to HDAC inhibition at 2-5 mM (Fig. 1D).

The study described here primarily involved colorectal cancer cell lines, and it will be important to confirm and extend these findings in gnotobiotic mouse models of colorectal cancer. For example, is it possible to manipulate the microbiota (by colonizing germfree mice with butyrate-producing bacteria) and diet (high fiber) to increase colonic butyrate levels and decrease tumorigenesis? If so, then it might useful to re-evaluate human epidemiologic studies that have had conflicting results regarding fiber consumption and colorectal cancer incidence. For example, might there be an association between participants' microbiota and whether or not a high-fiber diet protects against colorectal cancer? This is an important issue because the incidence of colorectal cancer has increased as we have shifted away from traditional, high-fiber diets towards processed foods that contain less complex carbohydrates and more refined sugars. To counteract this trend, probiotic and prebiotic strategies could possibly be implemented to increase the levels of an endogenous HDAC inhibitor (butyrate) in its normal environment (the colon). This chemoprevention strategy is likely to have fewer adverse effects than the i.v. delivery of synthetic HDAC inhibitors as chemotherapeutic agents (which are in clinical trials or have already received FDA approval, primarily for the treatment of hematopoietic malignancies).
